# Genotype‐phenotype relationships in mucopolysaccharidosis type I (MPS I): Insights from the International MPS I Registry

**DOI:** 10.1111/cge.13583

**Published:** 2019-07-02

**Authors:** Lorne A. Clarke, Roberto Giugliani, Nathalie Guffon, Simon A. Jones, Hillary A. Keenan, Maria V. Munoz‐Rojas, Torayuki Okuyama, David Viskochil, Chester B. Whitley, Frits A. Wijburg, Joseph Muenzer

**Affiliations:** ^1^ Department of Medical Genetics, B.C. Children's Hospital Research Institute University of British Columbia Vancouver British Columbia Canada; ^2^ Department of Genetics, Federal University of Rio Grande do Sul and Medical Genetics Service Hospital de Clínicas de Porto Alegre Porto Alegre Brazil; ^3^ Centre de Référence des Maladies Héréditaires du Métabolisme Hôpital Femme Mère Enfant Bron Cedex France; ^4^ Manchester Centre for Genomic Medicine Manchester University NHS Trust Manchester UK; ^5^ Sanofi Genzyme Cambridge Massachusetts; ^6^ Department of Clinical Laboratory Medicine National Center for Child Health and Development Tokyo Japan; ^7^ University of Utah Salt Lake City Utah; ^8^ Department of Pediatrics University of Minnesota Minneapolis Minnesota; ^9^ Experimental and Clinical Pharmacology University of Minnesota Minneapolis Minnesota; ^10^ Department of Pediatrics Academic Medical Center Amsterdam The Netherlands; ^11^ University of North Carolina at Chapel Hill Chapel Hill North Carolina

**Keywords:** genotype‐phenotype, hurler syndrome, iduronidase, lysosomal storage disease, lysosome, metabolic disease, mucopolysaccharidosis, Scheie syndrome

## Abstract

Mucopolysaccharidosis type I (MPS I) is a rare autosomal recessive disorder resulting from pathogenic variants in the α‐L‐iduronidase (*IDUA*) gene. Clinical phenotypes range from severe (Hurler syndrome) to attenuated (Hurler‐Scheie and Scheie syndromes) and vary in age of onset, severity, and rate of progression. Defining the phenotype at diagnosis is essential for disease management. To date, no systematic analysis of genotype‐phenotype correlation in large MPS I cohorts have been performed. Understanding genotype‐phenotype is critical now that newborn screening for MPS I is being implemented. Data from 538 patients from the MPS I Registry (380 severe, 158 attenuated) who had 2 *IDUA* alleles identified were examined. In the 1076 alleles identified, 148 pathogenic variants were reported; of those, 75 were unique. Of the 538 genotypes, 147 (27%) were unique; 40% of patients with attenuated and 22% of patients with severe MPS I had unique genotypes. About 67.6% of severe patients had genotypes where both variants identified are predicted to severely disrupt protein/gene function and 96.1% of attenuated patients had at least one missense or intronic variant. This dataset illustrates a close genotype/phenotype correlation in MPS I but the presence of unique *IDUA* missense variants remains a challenge for disease prediction.

## Graphical abstract

Mucopolysaccharidosis type I patient's variants and genotypes.

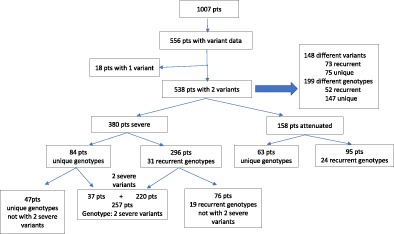

## 1 INTRODUCTION

Mucopolysaccharidosis type I (MPS I) is a rare recessive lysosomal storage disorder caused by pathogenic α‐L‐iduronidase (*IDUA*) gene variants which lead to deficiency of the lysosomal enzyme IDUA, EC 3.2.1.76.^1^ Historically, MPS I patients have been classified into three phenotypic disease categories; Hurler, Hurler‐Scheie and Scheie syndrome.^2^ This nosology was based on age of symptom onset and presence of progressive intellectual disability; with Hurler syndrome representing the most severely affected individuals, characterized by onset of symptoms in early infancy with evidence of early progressive intellectual decline and when untreated, death within the first decade. Hurler‐Scheie and Scheie syndromes represent later onset of disease symptoms, more slowly progressive disease with sparing of intelligence. Although Hurler syndrome represents a distinctive phenotype, Hurler‐Scheie and Scheie syndromes encompass a broad phenotypic spectrum with less precise delineation. From the practical perspective of disease management, a binary classification of patients into the categories of severe disease (Hurler syndrome) or attenuated disease (Hurler‐Scheie and Scheie syndromes) is most useful. This classification facilitates management decisions based on the currently approved treatments for MPS I: hematopoietic stem cell transplantation (HSCT) and enzyme replacement therapy (ERT) with laronidase.^3^ ERT has been shown to alter somatic disease symptoms and progression but as current recombinant enzyme does not cross the blood‐brain barrier this treatment approach does not address the progressive central nervous system (CNS) disease characteristic of Hurler patients.^4^ HSCT particularly when initiated early, has been shown to preserve cognition and lead to improved developmental outcomes and is thus considered the standard of care for patients with severe MPS I.^5^, ^6^ The recent initiation of newborn screening for MPS I as well as other programs to identify individuals with MPS I at an age when CNS and somatic involvement may be minimal, highlight the need for accurate delineation of patients so effective therapies can be initiated early. 7, 8, 9, 10, 11, 12 Unfortunately, no currently available biochemical assessments allow for accurate classification of patients as either severe or attenuated. 13 Published diagnosis and management guidelines suggest a role for IDUA genotype in management decision making.^7^ Over 200 pathogenic *IDUA* variants have been reported to underlie MPS I and a genotype‐phenotype association is emerging whereby patients who are either homozygous or compound heterozygous for the common nonsense mutations W402X or Q70X consistently have severe disease.^14^, ^15^, ^16^, ^17^, ^18^, ^19^ The large number of patients enrolled in the MPS I Registry offers a unique resource for further elaboration of genotype/phenotype relationships for this rare disease.^20^, ^21^, ^22^

## 2 MATERIALS AND METHODS

### 2.1 The MPS I Registry

The MPS I Registry (https://clinicaltrials.gov, NCT00144794) is a voluntary, long‐term, multinational, observational disease registry, established in 2003 to characterize the natural history of MPS I disease and track clinical outcomes.^21^, ^22^ Participating sites complete detailed enrollment forms including demographics, a multi‐domain medical history related to presence and onset of symptoms, and the type and starting date of any treatment. As participation in the Registry is voluntary, it is not known what proportion of individuals choose not to participate. Individuals may opt out of the Registry at any time. The Registry is sponsored by Sanofi Genzyme (Cambridge, Massachusetts) as part of the Food and Drug Administration (FDA) regulatory requirements for licensing of the ERT drug, laronidase. An independent board of advisors, comprised of clinicians with expertise in the care of patients with MPS I provides scientific oversight and direction of the Registry. Registry protocols and informed consent forms and procedures are reviewed and approved by local institutional review boards or independent ethics committees. All patients enrolled in the Registry have confirmation of MPS I diagnosis by either mutation analysis or measurement of IDUA levels. Written patient or parental consent is required for enrollment in the Registry.

### 2.2 Study population

Clinical and molecular genetic data of patients enrolled in the international MPS I Registry as of April 2017 were evaluated. Patients were included if they had (a) known diagnostic date and date of birth, (b) severity designation of Hurler, Hurler‐Scheie or Scheie as reported by the individual centers, (c) standardized *IDUA* genotype data with at least 1 *IDUA* sequence variant. Neither the laboratory method used to determine the sequence variants nor whether parental testing confirmed the inheritance or phase of the variants is captured by the Registry. In addition, the Registry does not capture known *IDUA* polymorphisms. A total of 1007 participants were enrolled in the MPS I Registry as of April 2017 with *IDUA* variant data available for 556 (55%). Those with available genotype data did not vary substantially from those who did not (n = 451). Eighteen patients had only a single variant reported with 538 having two reported variants. For genotype/phenotype associations, patients designated as Hurler were considered “severe” and patients designated as Hurler‐Scheie or Scheie were considered as “attenuated.” The terms “unique variants” and “unique genotypes” are defined as occurring in only one patient within this registry dataset and do not imply that the variant has not been previously published.

### 2.3 Statistical analyses

Continuous data are presented as means and SD or medians and interquartile ranges (25%, 75%). Categorical variables are presented as the number of observations and percentages n (%). Negative age values for diagnosis indicate prenatal diagnosis. All analyses were performed in Microsoft Excel 2010 and the Statistical Analysis System (SAS) 9.4 (Cary, North Carolina).

## 3 RESULTS

### 3.1 Patient demographics

Of the 556 eligible patients, 538 had two *IDUA* variants reported. Demographic information listing the geographic region of the Registry sites and basic diagnostic information on these 538 patients are listed in Table 1. The majority of patients are from European or North American sites. Latin America is under represented in this dataset as participants from this region are currently undergoing updated consenting. Among the eligible patients, 380 patients were classified as severe and 158 classified as attenuated. As expected, the mean age of diagnosis among severe patients (1.2 years) is at a younger age than attenuated (7.0 years).

**Table 1:** Patient demographics

Parameter	Severe N = 380	Attenuated N = 158	Total N = 538
Sex N (%)			
Male	190 (50)	73 (46)	263 (49)
Female	190 (50)	85 (54)	275 (51)
Geographic region N (%)			
Europe	219 (58)	105 (67)	324 (60)
North America	145 (38)	45 (29)	190 (35)
Latin America	11 (3)	5 (3)	16 (3)
Asia Pacific	5 (1)	3 (2)	8 (2)
Age at diagnosis (years)			
Mean (SD)	1.2 (1.6)	7.0 (8.7)	2.9 (5.6)
Min, max	0, 18.4	0, 54.1	0, 54.1

### 3.2 Statistical summary of variants and genotypes

**Figure 1:**
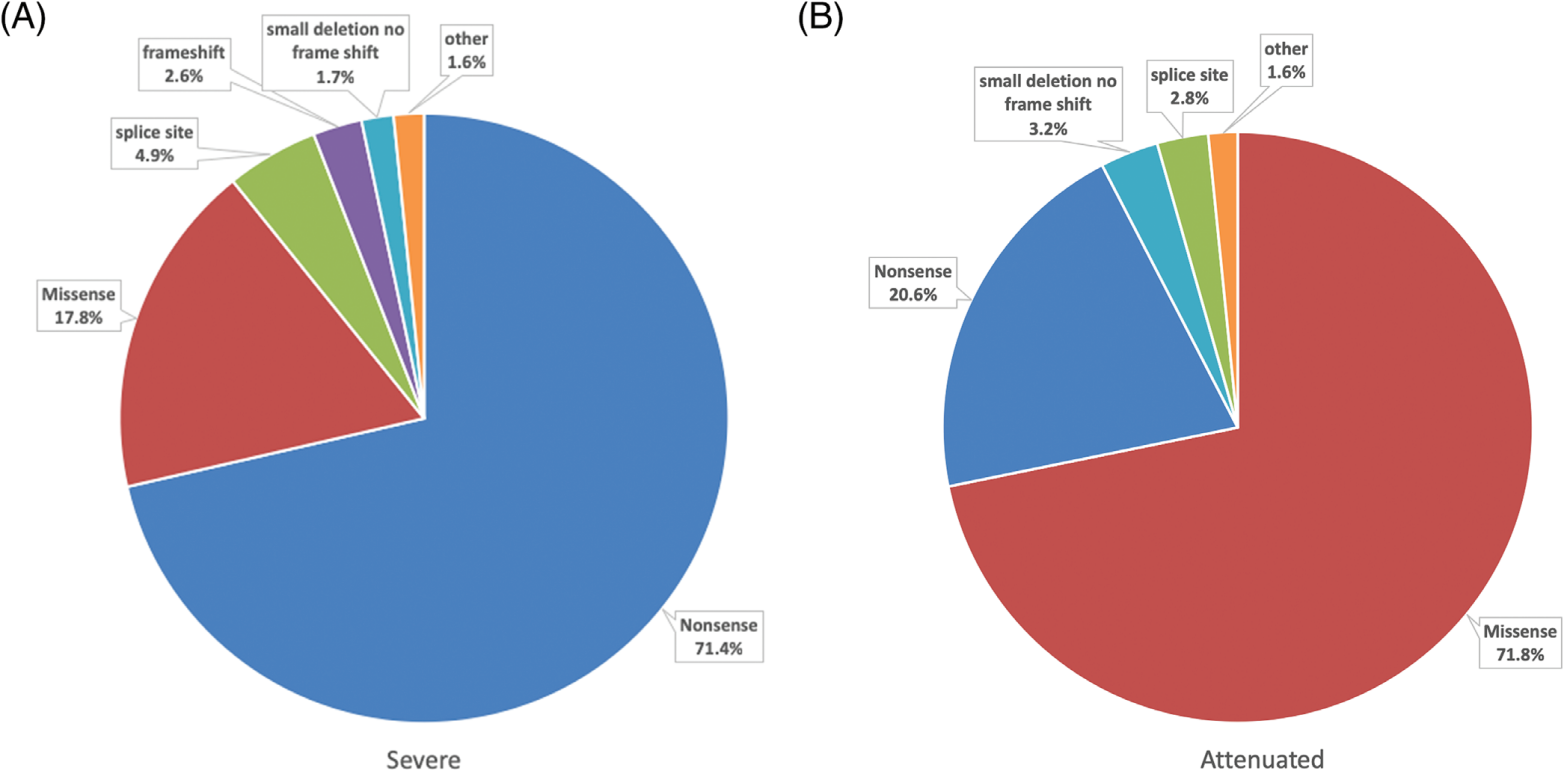
Distribution of variants in patients with two identified variants

A total of 1094 variants were reported among the 556 eligible patients, of these, 148 different *IDUA* variants were identified. Seventy‐four variants, representing 50% of all variants occurred only once. Eighteen patients (3%) had only one allele identified; 10 were classified as severe and eight classified as attenuated. Figure 1 and Table 2 depict the types and frequency of variants reported for the subtypes and entire cohort of the 538 patients with two reported variants. For severe patients (n = 380), nonsense variants were the most common (71.4%), followed by missense (17.8%), splice sites variants (4.9%) and frameshifts (2.6%). The most common variant among attenuated patients (n = 158) were missense (71.8%) followed by nonsense (20.6%), small deletions with no frame shift (3.2%) and splice site (2.8%).

**Table 2:** Summary of variant types

	Severe 380 patients alleles: n (%):	Attenuated 158 patients alleles: n (%)	Total 538 alleles: N (%)
Total alleles	760	316	1076
Nonsense	543 (71.4)	65 (20.6)	608 (56.5)
Missense	135 (17.8)	227 (71.8)	362 (33.6)
Splice site	37 (4.9)	9 (2.8)	46 (4.3)
Frameshift	20 (2.6)	1 (0.3)	21 (1.9)
Synonymous	0	2 (0.6)	2 (0.2)
Intronic	7 (0.9)	2 (0.6)	9 (0.8)
Large insertion	1 (0.1)	0 (0)	1 (0.1)
Initiator codon	4 (0.5)	0 (0)	4 (0.4)
Small deletion (no frameshift)	13 (1.7)	10 (3.2)	23 (2.1)

**Figure 2:**
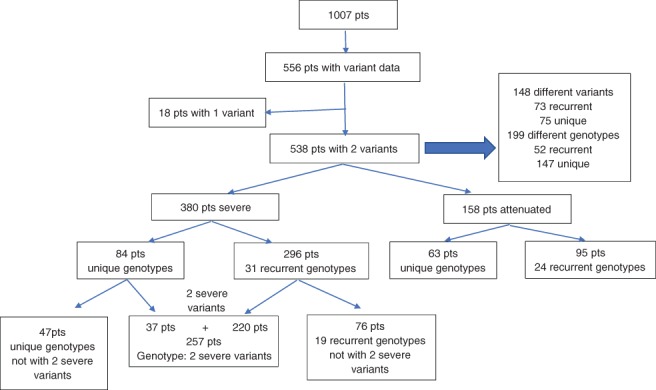
Patient's variants and genotypes [Colour figure can be viewed at http://wileyonlinelibrary.com]

In the 538 patients who had two alleles reported, 199 different genotypes were identified with 147 unique genotypes that is, occurring in only one individual (27.3% of all patients). In the patients, 39.9% (63/158) of attenuated patients and 22.1% (84/380) of severe patients had unique genotypes. A statistical summary of patients, variants and genotypes is depicted in Figure 2.

### 3.3 Genotype‐phenotype relationships

#### 3.3.1 Severe patients

There were 115 individual genotypes represented in the 380 patients with a severe phenotype and two reported alleles; 31 genotypes were recurrent and 84 genotypes unique (Table 3). The most common genotypes in severe patients were W402X/W402X (109/380; 28.7%), W402X/Q70X (61/380; 16.1%) and Q70X/Q70X (24/380; 6.3%). In total, these two nonsense variants defined the genotypes of 51% (194/380) of severe patients. Of severe patients, 67.6% (257/380) were either homozygous or compound heterozygous for two variants predicted to severely disrupt gene transcription or translation (eg, nonsense variants, frameshifts, consensus splice site disruption or initiator codon variants). No clinically diagnosed attenuated patients were homozygous or compound heterozygous for such variants. Of the remaining 123 severe patients, 76 comprised 19 recurrent genotypes (Table 3) with the remaining 47 patients (12.6%) having unique genotypes (listed in Table S1). Thirty‐seven of the 84 unique genotypes identified in severe patients were compound heterozygous with two severe variants (as defined above) and were comprised of 34 separate severe variants (listed in Table S1). There were nine missense recurrent variants and one intronic recurrent variant that were exclusively seen in severe patients (Table 5A).

**Table 3:** Severe MPS I patients: N = 380

Two severe^a^ variants	Recurrent genotypes^b^		Unique genotype^c^
257 (67.6%)	296 (77.9%)		47 (12.4%)
	W402X/W402X	109 (28.7%)	
	W402X/Q70X	61 (16.1%)	
	Q70X/Q70X	24 (6.3%)	
	R628X/R628X	6 (1.6%)	
	p.S16_A19del/W402X	6 (1.6%)	
	R619X/W402X	5 (1.3%)	
	p.P21fs/W402X	3 (0.8%)	
	K153X/W402X	2 (0.5%)	
	c.386‐2A > G/W402X	2 (0.5%)	
	c.386‐2A > G/c.386‐2A > G	2 (0.5%)	
	c.158 + 1G > A/W402X	2 (0.5%)	
	c.1524 + 1G > T/W402X	2 (0.5%)	
	c.1403‐1G > T/Q400X	2 (0.5%)	
	A327P/W402X	14 (3.7%)	
	A327P/Q70X	5 (1.3%)	
	A327P/A327P	3 (0.8%)	
	P533R/P533R	7 (1.8%)	
	P533R/W402X	5 (1.3%)	
	P533R/R363L	2 (0.5%)	
	P533R/D301E	2 (0.5%)	
	L218P/Q70X	5 (1.3%)	
	L218P/W402X	3 (0.8%)	
	L218P/L218P	2 (0.5%)	
	A75T/Q70X	2 (0.5%)	
	A75T/W402X	2 (0.5%)	
	T388R/W402X	5 (1.3%)	
	P496R/Q70X	4 (1.1%)	
	c.1650 + 5G > A/W402X	3 (0.8%)	
	G51D/G51D	2 (0.5%)	
	L18P/T388K	2 (0.5%)	
	N110D/Q70X	2 (0.5%)	

a Any combination of nonsense, consensus splice site, initiator codon or frame shift.

b Including genotypes comprising two severe variants.

c Occurring in only one individual and exclusive of two severe alleles (see Tables S1 and S2).

#### 3.3.2 Attenuated patients

There were 87 individual genotypes represented in the 158 patients with an attenuated phenotype and two reported alleles; 24 genotypes were recurrent in a total of 95 patients (60%) and 63 unique (40%) (Table 4 and Table S2). The most common genotypes in attenuated patients were L490P/L490P (21/158; 13.3%), P533R/P533R (17/158; 10.8%) and L238Q/W402X (6/158; 3.8%). 46.8% of attenuated patients (74/158) were heterozygous for a variant predicted to be severely disruptive to gene transcription or translation (as defined above). Within that patient cohort, 97.5% (154/158) of attenuated patients had at least one missense variant representing 95.4% (83/87) of the genotypes. The remaining four genotypes were: p.D445del/W402X, c.386‐2A > G/c.590‐7G > A, p.S16_A19del/X654R and p.S16_A19del/p.S16_A19del. A total of 54 independent missense variants were identified within the attenuated patients and 23 of these variants were recurrent (homozygous alleles counted as a single occurrence). Fifteen of the 23 recurrent missense variants identified in this patient cohort were unique to attenuated patients (Table 5B). Two variants involving the canonical translation stop codon X654; X654G and X654R were unique to attenuated patients and contained within the following genotypes; X654G/Q70X (one patient), X654R/p.S16_A19del (two patients), X654R/Q380X (1 patient). There were two attenuated patients who were compound heterozygous with synonymous variants in combination with W402X. One variant, K264K, is at the terminal amino acid of exon II and thus may alter splicing, the other variant N297 N is contained within exon VII. Both of these variants are thus variants of unknown significance.

**Table 4:** Attenuated MPS I patients: N=158

Missense variants^a^	Recurrent genotype^b^		Unique genotype^c^
154 (95.4%)	95 (60%)		63 (40%)
	L490P/L490P	21 (13.3%)	
	P533R/P533R	17 (10.8%)	
	P533R/W402X	3 (1.9%)	
	P533R/Q70X	2 (1.3%)	
	L238Q/W402X	6 (3.8%)	
	L238Q/Q70X	2 (1.3%)	
	Q380R/Q380R	4 (2.5%)	
	Q380R/T388R	2 (1.3%)	
	Q380R/W402X	2 (1.3%)	
	R383H/ c.386‐2A > G	3 (1.9%)	
	R383H/W402X	2 (1.3%)	
	R383H/Q70X	2 (1.3%)	
	R89Q/W402X	4 (2.5%)	
	R89W/W402X	3 (1.9%)	
	S633 L/p.S16_A19del	3 (1.9%)	
	S633 L/W402X	3 (1.9%)	
	p.S16_A19del/E178K	2 (1.3%)	
	A327P/A327P	2 (1.3%)	
	A36E/Q70X	2 (1.3%)	
	G265R/W402X	2 (1.3%)	
	L535F/W402X	2 (1.3%)	
	N348K/W402X	2 (1.3%)	
	Q380X/X654R	2 (1.3%)	
	c.1727 + 5G > C/N348K	2 (1.3%)	

a Genotype contains at least one missense.

b Occurring in greater than or equal to two patients.

c See Tables S1 and S2.

**Table 5:** Phenotype exclusive recurrent variants (recurrent = ≥ two patients)

Variant	Patients	Homozygous	Compound with severe	Compound other
A: Missense and intronic^a^ variants exclusive to severe patients				
A75T	5	1	4	0
G208D	3	0	1	2
D301E	2	0	0	2^†^
D301H	2	0	1	1^††^
D349N	2	0	1	1
N110D	2	0	2	0
R363L	2	0	0	2^†^
T388K	2	0	0	2^†††^
M1L	2	1	0	1^††††^
c.1650 + 5G > A	4	1	3	
B: Exclusive to attenuated patients				
L490P	21	21	0	0
Q380R	13	4	5	4
S633 L	10	1	3	6
R383H	9	0	7	2
R89Q	7	0	5	2
R89W	6	1	3	2
N348K	4	0	2	2^b^
G265R	3	0	2	1
H240R	3	0	2	1
S347R	2	1	1	0
A36E	2	0	2	0
E178K	2	0	0	2
L535F	2	0	2	0
P510R	2	0	2	0
R505G	2	0	2	0
c.1727 + 5G > C	2	0	0	2^b^
X654R	3	0	1	2

^†^ P533R,

^††^ D349Y,

^†††^ L18P,

^††††^ S443R.

^a^ Not contained within consensus splice sites.

^b^ Two patients N348K/c.1727 + 5G > C.

### 3.4 Genotypes and variants of special interest

#### 3.4.1 P533R

P533R was found in a total of 46 patients as either homozygous or compound heterozygous with other alleles (Table 6A). A total of 24 patients were homozygous for P533R; seven classified as severe and 17 classified as attenuated. An additional 12 patients were compound heterozygous for P533R and a recurrent nonsense allele with five classified as severe and seven attenuated.

**Table 6:** Variants of special interest

Genotype	Patients	Severe	Attenuated
A: P533R variant containing genotypes (bolded found exclusive to attenuated patients)			
P533R/P533R	24	7	17
P533R/W402X	8	5	3
P533R/Q70X	2	0	2
P533R/R621X	1	0	1
P533R/K153X	1	0	1
P533R/p.H425fs	1	1	0
P533R/R363L	2	2	0
P533R/D301E	2	2	0
P533R/**S633 L**	1	0	1
P533R/**R89Q**	1	0	1
P533R/I259N	1	0	1
P533R/G197D	1	0	1
P533R/D184V	1	0	1
B: A327P variant containing genotypes (bolded found exclusive to attenuated patients)			
A327P/A327P	5	3	2
A327P/W402X	14	14	0
A327P/Q70X	5	5	0
A327P/c1190‐1G > C	1	1	0
A327P/**Q380R**	1	0	1
A327P/**R383H**	1	0	1
A327P/S423R	1	1	0
A327P/**R89W**	1	0	1
A327P/T374 N	1	0	1

#### 3.4.2 A327P

The A327P variant was identified in a total of 30 patients (Table 6B). Five patients were homozygous for this variant three classified as severe and two attenuated. All 20 of the patients where A327P was compound heterozygous with a severe allele were classified as severe.

#### 3.4.3 L18P

The L18P variant was found in a compound heterozygous state in five patients; two severe and three attenuated. Two of the attenuated patients were compound heterozygous with the recurrent nonsense variants W402X and Q70X with the other compound with the recurrent missense variant P496R. The two severe patients compound heterozygous for L18P had the missense variant T388K as their other variant. These are the only patients with the T388K variant.

#### 3.4.4 P496R

The P496R variant was noted in seven patients; six severe and one attenuated. The single attenuated patient was compound heterozygous with L18P and the severe patients were compound with Q70X (n = 4), c.1727 + 1 G > A (n = 1) or homozygous (n = 1). P496R appears to lead to severe disease when homozygous or compound heterozygous with other recurrent nonsense variants as demonstrated with the Q70X variant.

#### 3.4.5 Deletions within the signal peptide

**Figure 3:**
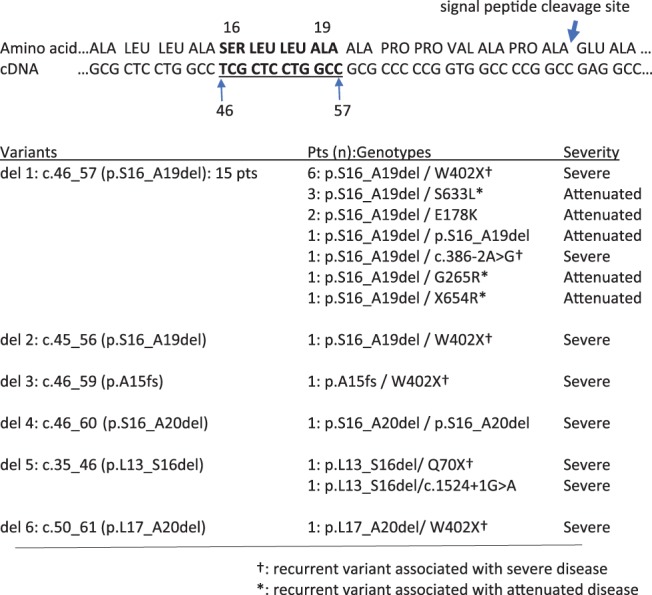
Patients with deletions of signal peptide region [Colour figure can be viewed at http://wileyonlinelibrary.com]

Twenty‐one patients had deletions within the signal peptide region as designated in Figure 3. The out‐of‐frame deletion, c.46_59 (p.A15fs) was noted in one individual with severe disease and was compound heterozygous with the common nonsense variant W402X. Fifteen patients had the in‐frame deletion c.46_57 (p.S16_A19del), seven were classified as severe, each being heterozygous for recurrent variants associated with severe disease; six with W402X and one with c.3.386‐2A > G. The remaining eight patients with c.46_57 (p.S16_A19del) were attenuated; one homozygous, five were compound heterozygous for recurrent variants associated with attenuated disease (S633 L, G265R and X654R), and two heterozygous for E178K (the only patients with E178K). Five patients had each of the in‐frame deletions, c.45_56 (p.S16_A19del), c.46_60 (p.S16_A20del), c.35_46 (p.L13_S16del) or c.50_61 (p.L17_A20del) and were severe. One patient was homozygous for p.S16_A20del and the others were compound with severe the alleles; W402X, Q70X or c.1524 + 1G > A.

## 4 DISCUSSION

The genotype/phenotype relationships in this extensive series of 538 MPS I patients confirms and extends earlier observations that a large portion of severe MPS I patients (67.6%) have two *IDUA* variants that would be predictive of null alleles. Consequently, these data show that homozygosity or compound heterozygosity for two severe variants always leads to severe disease. In this series of Registry patients, none of the 158 attenuated patients had genotypes where both variants would predict severe disruption of transcription or translation; however, 95.6% (151/158) of attenuated patients had at least one missense variant with the remainder having at least one splice site, intronic or small in‐frame deletion variant. As expected in a recessive disorder, this Registry's genotype/phenotype data indicates that attenuation of MPS I disease results from at least one *IDUA* missense allele which must confer residual IDUA enzyme activity, thereby emphasizing the importance of identifying both pathogenic alleles in patients to accurately and effectively utilize genotype to guide patient management. Although these observations and generalizations are clear, from a practical perspective of the observation that 40% of patients with attenuated disease had unique genotypes and 12.4% of severe patients had unique genotypes not containing two predictably severe variants illustrates the complexity of solely using genotype to predict phenotype in all patients. This is compounded by the observation that certain common missense variants appear to be associated with variable phenotypes.

This Registry's data highlight the important role that missense variants and much less commonly, intronic variants, play in disease attenuation and identifies certain recurrent missense variants and intronic variants that are consistently associated with either severe or attenuated disease (Table 5). The genotypes and respective phenotypes associated with these recurrent missense variants within this Registry dataset should help to guide the management of a significant portion of patients with MPS I.

The large number of patients in the MPS I Registry shows both the benefit of genotypes to guide clinical care, as well as the complexity of understanding these genetic variants. Care must be taken in interpreting the predictive value of each missense variant as many occur on rare genotypes. In addition, these Registry data also illustrate that certain missense variants appear to have variable effects (Table 6A,B ) even when homozygous. This is highlighted by the variable phenotypes seen in patients homozygous for the variants P533R and A327P, as well as patients with L18P, P496R and variants within the signal peptide region. There were 46 patients with the P533R variant, 24 of whom were homozygous yet, seven of the 24 were classified as severe and 17 attenuated. Thirteen patients were compound heterozygous for P533R and a predictably severe variant (as described above) yet six were classified as severe and seven attenuated. Of the remaining nine P533R patients, four were classified as severe and the five classified as attenuated had other missense variants with only two recurrent variants, R89Q and S633 L that are associated with attenuated disease within this dataset. These observations are contrasted by the 30 patients with the A327P variant. In 19 patients, the A327P variant was compound heterozygous with the common severe variants W402X (n = 14) and Q70X (n = 5) and all 19 patients were classified as severe. Three of the five homozygous A327P patients were classified as severe and two were classified as attenuated. Three of the remaining six patients had recurrent variants that are associated with attenuated disease (Q380R, R383H and R89W) and are classified as attenuated. The genotypes of the remaining three patients had A327P associated with the following variants: c.1190‐1 G > C, severe; S423R, severe; and T374 N, attenuated. Functional and structural studies of the P533R variant have shown that the *K*_m_ for artificial IDUA substrates is not perturbed by this variant but the *V*_max_ is decreased by 2‐fold compared to wildtype IDUA. In addition, the P533R mutant protein showed lower thermodynamic stability suggesting the potential of impaired processing and trafficking. No functional studies have been performed on the A327P variant.

Even though the crystal structure of IDUA complexed with various substrates has been published, the conformation of the active site defined, and it has been shown that the high mannose glycan at Asn372 contributes to the enzymatic activity, it still remains a challenge to use this structure directly to a priori predict the functional effect of sequence variants on the processing and enzymatic activity of the protein.^23^, ^24^, ^25^, ^26^ Most of the recurrent variants reported here have been discussed in relation to in silico structural and functional effects on the IDUA protein and although one can use these various models to explore the effects of variants on protein structure, the real‐life data reported through the Registry is invaluable and arguably more relevant to clinical management decisions. Enzyme measurement of IDUA activity is not helpful as unfortunately, currently available substrates used for the measurement of white cell, plasma or fibroblast iduronidase enzyme activity does not allow for accurate classification of patients.

With the establishment of newborn screening and initiatives to diagnose MPS I patients at a younger age, the current genotype/phenotype observations from the MPS I Registry emphasize the urgent need for the establishment of an international open‐access curated database of *IDUA* sequence variants, if not complete *IDUA* genomic sequence data, linked to basic clinical disease severity scoring of MPS I patients. The availability of the entire *IDUA* genomic sequence from MPS I patients would be essential for elucidating the role *IDUA* polymorphisms may play in modulating the effects of pathogenic *IDUA* variants. This could provide a better understanding of factors either allelic or non‐allelic which may underlie the clinical variability of certain variants. Within this current series of MPS I patients, none were reported to have any of the know *IDUA* pseudodeficiency variants; A79T, H82Q, V322E or D223N.^27^ However, we cannot be certain that individuals did not have these variants in addition to the two presumed pathogenic variants reported. Nevertheless, the lack of these variants confirms that no MPS I patients within the Registry appear to be IDUA pseudodeficient.

There are a number of limitations to this Registry‐based study. The variants reported are submitted by the individual centers and data are not collected to ascertain whether parental studies have confirmed the phase of the variants, the laboratory classification of the status of the variants, what methodology was used to identify the variant or whether the variants have been submitted to any variant database. Thus, the variants reported here are not curated. In addition, the classification of patients as severe (Hurler) or attenuated (Hurler‐Scheie or Scheie) is left to the submitting physician. As no biochemical methods are known to distinguish the phenotypic spectrum of MPS I the clinical classification of patients could not be directly validated in this study.

This large Registry dataset illustrates that there is indeed a close genotype/phenotype correlation in MPS I and that for the majority of patients, phenotype can be accurately predicted from the genotype alone. This does not mean that one can necessarily accurately predict the phenotype from any genotype, as the precise effect of unique sequence variants, particularly unique missense variants on the processing and catalytic properties of the enzyme are often not known. The genotype/ phenotype relationships identified in this large series of patients shows that many MPS I patients have unique variants and that certain variants have variable phenotypic effects. The analyses in this study have used a binary classification of patients into severe and attenuated. Although from a practical perspective of management decision making this nosology is helpful, from a phenotype perspective this is likely an over simplification as attenuated patients definitely fit a broader phenotypic spectrum than severe patients. These observations underscore the need for development and evaluation of biomarkers, more comprehensive genomic analysis and cellular expression studies that could be integrated into the evaluation of patients.

## CONFLICT OF INTEREST

L.A.C. is a member of the MPS I Registry advisory board (supported by Sanofi Genzyme) and receives speaker's fees for educational events related to lysosomal disease from Sanofi.

R.G. is honorarium from Sanofi Genzyme for expert advice, travel grants from Sanofi Genzyme to attend scientific meetings, investigator fees from Sanofi Genzyme, unrestricted grants from Sanofi Genzyme to promote educational events. N.G. is advisory boards for Sanofi Genzyme and participated in MPS I Registry. S.A.J. has received travel assistance and consultancy fees for Sanofi Genzyme. H.A.K. is an employee of Sanofi Genzyme. J.M. serves on advisory boards and consult for Sanofi Genzyme and BioMarin. M.V.M.‐R. is an employee of Sanofi Genzyme. T.O. receives a research grant from Sanofi Genzyme and has attended advisory boards for Sanofi Genzyme. D.V. is honoraria for speaking, consulting, and travel expenses from Sanofi Genzyme.

Dr. C.B.W. cites clinical trial contract work from Sanofi‐Genzyme. F.A.W. none to disclose.

## Supporting information


**Table S1.** Unique genotypes amongst severe patients
**Table S2.** Unique genotypes amongst attenuated patients

## Data Availability

The data that support the findings of this study can be requested by MPS I Registry participants through a MPS I Registry Data Analyses Request form. The data are not publicly available due to privacy or ethical restrictions. For additional information, please contact rarediseaseregistries@sanofi.com
