# Supplementary material for: Genotype‐phenotype relationships in mucopolysaccharidosis type I (MPS I): Insights from the International MPS I Registry
Source: Clin Genet. 2019 Jul 2;96(4):281–9. doi: 10.1111/cge.13583 (PMC6852151; doi:10.1111/cge.13583)
Supplement: Supplementary file 1 — Table S1. Unique genotypes amongst severe patients Table S2. Unique genotypes amongst attenuated patients [file CGE-96-281-s001.docx]

**Supplemental Tables:**

**Table S1; Unique Genotypes Amongst Severe Patients**

| **Compound with 2 severe variants** | **Compound with 1 severe variant*** | **Other** |
| --- | --- | --- |
| p.S347fs/p.S347fs | p.G219fs/A319E | A327P/S423R |
| p.F495fs/p.F495fs | c.1190-1G>C/A327P | A75T/A75T |
| p.S600X/p.S600X | W402X /A351P | c.1650+5G>A/c.1650+5G>A |
| p.A15fs/W402X | c.386-2A>G/A79V | D301H/D349Y |
| M1L/M1L | p.H425fs/P533R | E182K/G208D |
| Q407X/W402X | p.A8fs/D624V | G208D/N350K |
| Q561X/W402X | c.1727+1G>A/P496R | G208V/G208V |
| Q60X/Q70X | W306X /c.1727+5G>A | L439P/L439P |
| Q70X/R628X | c.386-2A>G/L14R | P496R/P496R |
| Q70X/Y167X | c.386-2A>G/L218P | c.159-19_160del/D349N |
| Q70X/Y343X | c.386-2A>G/L238Q | p.S16_A20del/p.S16_A20del |
| R619X/R619X | c.590-1G>A/D301H |  |
| R621X/R621X | C53X/L218P |  |
| R621X/W402X | Q70X/D315Y |  |
| W402X/Y167X | W402X /D349N |  |
| W402X/Y471X | Q70X/G208D |  |
| W402X/Y581X | W402X/G51D |  |
| Y64X/Y64X | R619X/G84R |  |
| p.T388fs/W402X | W402X /H82P |  |
| c.1190-1del/Q70X | W402X /M504R |  |
| p.P6fs/W402X | Q70X/X653G |  |
| p.V538fs/Q70X | W402X /R363C |  |
| p.V538fs/W402X | W402X /R492P |  |
| c.1651-1G>A/Q70X | R619X/T364R |  |
| c.1728-1G>C/Q70X | R619X/T388R |  |
| c.1728-1G>C/W402X | R621X/T388R |  |
| p.S59fs/Q70X | W402X/R636C |  |
| p.D69fs/W402X | M1L/S443R |  |
| c.386-2A>G/Q70X | M1T/T364M |  |
| c.494-1G>A/W402X | Q70X/Y76C |  |
| c.792+1G>A/Q70X | p.T388fs/G51D |  |
| c.1403-1G>T/c.1728-1G>C | p.L13_S16del/Q70X |  |
| c.158+1G>A/c.158+1G>A | c.1524+1G>A/p.L13_S16del |  |
| c.1828+1G>A/c.1828+1G>A | c.386-2A>G/p.S16_A19del |  |
| c.386-2A>G/p.W180X | p.S16_A19del/W402X |  |
| c.494-1G>A/c.494-1G>A | p.L17_A20del/W402X |  |
| Q70X/Y202X |  |  |
|  |  |  |

**TABLE S2: Unique Genotypes Amongst Attenuated Patients**

| A319V/A319V |
| --- |
| A327P/Q380R |
| A327P/R383H |
| A327P/R89W |
| A327P/T374N |
| A556D/S633L |
| C577Y/W402X |
| D184V/P533R |
| D203H/Q70X |
| D203N/W402X |
| D315Y/L218P |
| D349Y/Q380R |
| D484A/L564P |
| E182K/L238Q |
| G197D/P533R |
| G51D/R89W |
| H240R/T388R |
| H240R/Y581X |
| H33Q/W626X |
| I259N/P533R |
| K153X/P533R |
| K264K/W402X |
| L18P/P496R |
| L18P/Q70X |
| L18P/W402X |
| L209R/L209R |
| L238P/W402X |
| L396P/L396P |
| L526P/W402X |
| N297N/W402X |
| P496L/W402X |
| P533A/P533A |
| P533L/S633L |
| P533R/R621X |
| P533R/R89Q |
| P533R/S633L |
| Q380R/Q70X |
| Q380R/R621X |
| Q500R/W402X |
| Q563P/Q70X |
| Q70X/R89Q |
| Q70X/T374I |
| Q70X/X654G |
| R383H/R89Q |
| R435G/W402X |
| R505G/W152X |
| R505G/W402X |
| R89W/R89W |
| S347R/S347R |
| S347R/W402X |
| S423R/S423R |
| S586F/S586F |
| S633L/S633L |
| c.1189+1G>A/Q380R |
| p.D445del/W402X |
| c.1525-2A>C/P510R |
| c.1727+4C>T/G51D |
| c.386-2A>G/c.590-7G>A |
| p.S16_A19del/G265R |
| p.S16_A19del/X654R |
| p.S16_A19del/p.S16_A19del |
| p.P21fs/H240R |
| c.972+1G>A/P510R |
